# Spatial Patterns and Ecological Drivers of Sedimentary Eukaryotic Microorganisms Across Typical Depositional Zones of Lake Taihu

**DOI:** 10.3390/microorganisms14051121

**Published:** 2026-05-15

**Authors:** Zhendong Li, Yang Chen, Yajie Li, Aidong Ruan

**Affiliations:** College of Hydrology and Water Resources, Hohai University, Nanjing 210098, China; 231301020003@hhu.edu.cn (Z.L.); 240401010002@hhu.edu.cn (Y.C.); hhulyj@hhu.edu.cn (Y.L.)

**Keywords:** Lake Taihu, freshwater sediments, eukaryotic microorganisms, 18S rRNA gene sequencing, community assembly, co-occurrence networks, environmental drivers

## Abstract

Although sedimentary zones in Lake Taihu differ in external inputs, hydrodynamic conditions, and sedimentary settings, the spatial differentiation of eukaryotic microbial communities and their assembly mechanisms remain insufficiently understood. This study analyzed sediment cores from four typical sedimentary zones of Lake Taihu: Dapu (DP), Gonghu (GH), the central lake area (HX), and Xuhu (XH). By integrating physicochemical measurements, 18S rRNA gene high-throughput sequencing, redundancy analysis, functional annotation, iCAMP, and co-occurrence network analysis, we characterized the composition, environmental associations, and assembly mechanisms of sedimentary eukaryotic microbial communities. The results showed that eukaryotic microbial communities in Lake Taihu sediments exhibited marked spatial heterogeneity, with dominant taxonomic groups including Chlorophyta, Intramacronucleata, and Diatomea. Alpha diversity was higher in the GH zone and lower in the HX zone, whereas beta diversity showed significant separation among lake zones. NH_4_^+^-N, NO_3_^−^-N, TN, TP, TOC, D50, MWC, and pH were associated with variation in community composition, but the main associated factors differed among zones. FunGuild annotation showed that annotated fungal functional groups exhibited distinct trophic distribution patterns across sedimentary zones. iCAMP analysis indicated that community assembly was generally dominated by stochastic processes, with dispersal limitation prevailing in the GH zone and ecological drift dominating in the DP, HX, and XH zones. Co-occurrence network analysis further revealed marked differentiation in potential biological associations among sedimentary zones. Overall, this study showed that nutrient conditions and sediment physical properties in different sedimentary environments of Lake Taihu jointly shaped the spatial patterns of eukaryotic microbial communities and their ecological associations, providing baseline information for understanding sedimentary ecological processes in eutrophic shallow lakes.

## 1. Introduction

Lake Taihu is one of the five largest freshwater lakes in China. The Lake Taihu Basin supports a population of 160.33 million and contributes 18.1–18.8% of China’s GDP, while the lake itself serves as a critical water source for the Yangtze River Delta [1]. In recent years, however, increasing pollution loads have promoted recurrent algal blooms and threatened ecosystem stability in Lake Taihu [2]. Lake sediments are key interfaces for biogeochemical cycling; they function as nutrient “source-sink” regulators, and sediment resuspension can strongly affect the ecological status of the overlying water column [3]. Therefore, elucidating sedimentary ecological processes is essential for understanding and mitigating ecological degradation in Lake Taihu.

Microbial communities are key drivers of elemental biogeochemical cycling in sediments, and eukaryotic microorganisms, including fungi, protozoa, and microalgae, play essential roles in material transformation and energy flow [4,5]. Previous studies have shown that fungi often represent a substantial proportion of sedimentary eukaryotic communities and participate extensively in organic matter degradation, carbon fixation, methane cycling, and phosphorus transformation [6,7,8,9]. Moreover, eukaryotic microorganisms can regulate the stability and functioning of lake ecosystems by producing bioactive secondary metabolites, such as phenols, quinones, terpenoids, alkaloids, and peptides, and by contributing to food-web maintenance [10,11,12]. Lake Taihu is a shallow lake with distinct sedimentary zones shaped by hydrological, environmental, and lake-morphological conditions [13,14]. These zones include inflow-affected agricultural sediments in DP, urban-influenced sediments in GH, downstream and outflow-related sediments in XH, and open-lake central sediments in HX, which differ in external inputs, hydrodynamic exchange, depositional conditions, and sediment physicochemical properties. Such sedimentary contrasts may lead to spatial heterogeneity in resident eukaryotic microbial communities and their ecological functions. However, compared with bacteria and archaea, eukaryotic microorganisms in Lake Taihu sediments have received relatively limited attention, particularly with respect to their community structure, diversity patterns, and ecological functions across different lake regions [15,16,17].

From the perspective of ecological mechanisms, microbial community formation and succession are jointly governed by deterministic processes, including environmental selection and biotic interactions, and stochastic processes, including dispersal limitation and ecological drift [18,19,20]. However, the relative contributions of these mechanisms to the assembly of eukaryotic microbial communities in lake sediments remain unclear [21]. Microbial interactions can also be characterized using co-occurrence networks; however, most existing studies have focused on bacteria or individual microbial groups, leaving synergistic and antagonistic relationships among multiple eukaryotic groups, such as fungi, algae, and protozoa, insufficiently explored [22,23]. Notably, fungi and algae can form microecological associations around algal cells, such as the phycosphere, in freshwater systems, indicating that their complex ecological interactions require further investigation [24,25].

Against this background, this study investigated different sedimentary zones of Lake Taihu. Using sediment core sampling, environmental factor analysis, and 18S rRNA gene high-throughput sequencing, we systematically analyzed the structure and drivers of sedimentary eukaryotic microbial communities. Specifically, this study aimed to (1) reveal the composition, diversity, and spatial differentiation of eukaryotic microbial communities in different sedimentary environments and identify key environmental drivers; (2) infer their potential roles in ecological processes, such as carbon and nitrogen cycling, based on functional analysis; and (3) quantify the relative contributions of deterministic and stochastic processes to community assembly and characterize multikingdom co-occurrence network structures and potential ecological interactions. This study provides insights into the spatial patterns, assembly mechanisms, and interaction networks of eukaryotic microbial communities in Lake Taihu sediments, thereby supporting a deeper understanding of their ecological functions, potential responses to environmental change, and implications for lake ecosystem conservation and management. From an applied perspective, identifying zone-specific microbial indicators and their environmental drivers can help improve sediment ecological assessment, support differentiated monitoring of eutrophic shallow lakes, and provide biological evidence for sediment pollution control and water-quality management.

## 2. Materials and Methods

### 2.1. Sample Collection and Processing

**Figure 1:**
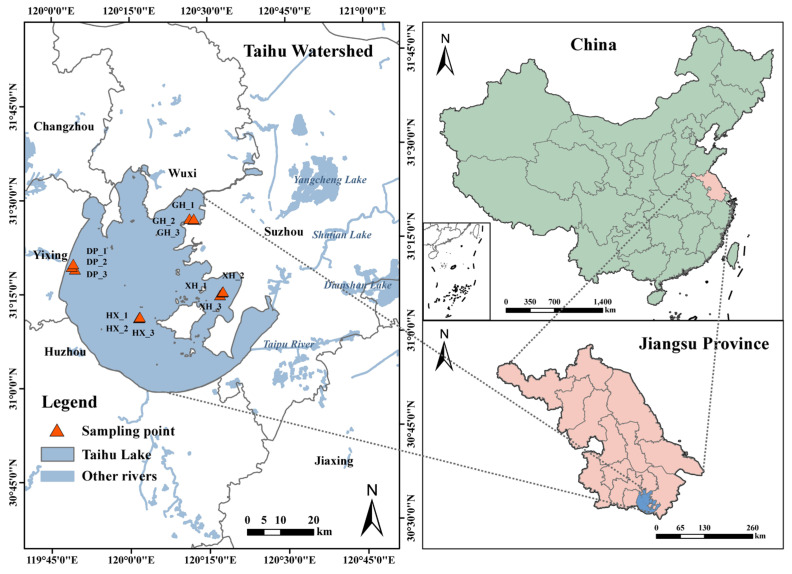
Sampling locations in Lake Taihu.

Based on the geographical, hydrological, and environmental characteristics of Lake Taihu, four study areas were selected: Dapu (DP), Gonghu (GH), Xuhu (XH), and central Lake Taihu (HX). Three replicate sampling sites were established within each selected area, resulting in a total of 12 sampling sites (Figure 1, Table S1). Among these areas, DP, located in western Lake Taihu and surrounded by farmland, receives inflows from five rivers and serves as a catchment for agricultural pollutants; GH, located in northern Lake Taihu adjacent to Wuxi City, receives inflows from two rivers and is influenced by urban pollutant inputs; and XH, located in southeastern Lake Taihu adjacent to Suzhou City, represents the downstream and outflow area of Lake Taihu. HX is located in the central lake area and is subject to relatively limited direct human disturbance. In December 2017, one sediment core was collected from each replicate sampling site using a gravity corer [18], yielding a total of 12 sediment cores. Each core covered a depth of 0–40 cm and was sectioned at 5 cm intervals into eight layers, resulting in 96 sediment samples. No radiocarbon dating was performed in this study. Therefore, sediment depth was used to represent vertical sedimentary microhabitat variation rather than sediment age or chronological sequence. After impurities and benthic organisms were removed, the samples were gently homogenized and then allocated into plastic bags, cryovials, and centrifuge tubes for subsequent analyses. Samples in plastic bags were frozen at −20 °C for physicochemical analysis, whereas samples in cryovials were freeze-dried for 72 h, transferred to centrifuge tubes, and stored at −80 °C in an ultra-low-temperature freezer for molecular ecological analysis.

### 2.2. Measurement of Sample Physicochemical Parameters

The physicochemical parameters measured for each sediment sample included ammonium nitrogen (NH_4_^+^-N), nitrate nitrogen (NO_3_^−^-N), total nitrogen (TN), total phosphorus (TP), total organic carbon (TOC), mass water content (MWC), median particle size (D50), and pH. A portion of each freeze-dried sediment sample was passed through a 2 mm sieve, ground, and then passed through a 100-mesh (0.154 mm) sieve for subsequent analyses. For pH determination, 1 g of the sieved sample was mixed with 2.5 mL of deionized water, shaken for 30 s, allowed to stand for 30 min, and measured using a portable pH meter (testo 206-pH2, Testo SE & Co. KGaA, Titisee-Neustadt, Germany) [26]. Nitrogen species were extracted from the sieved samples using KCl solution [27]. After filtration through a 0.45 μm membrane, NH_4_^+^-N and NO_3_^−^-N concentrations were measured using a continuous-flow water quality analyzer (SAN++, SKALAR, Breda, The Netherlands). TN, TP, and TOC were determined using a UV spectrophotometer (UV-1200B, MAPADA, Shanghai, China). For TN determination, the sieved samples were digested with potassium persulfate, and the absorbance of the digest was measured at 220 and 275 nm. For TP determination, samples were digested with HNO_3_-HF-HClO_4_, color development was performed using the molybdenum blue method, and absorbance was measured at 700 nm. For TOC determination, organic carbon was oxidized using K_2_Cr_2_O_7_-H_2_SO_4_, and absorbance was measured at 585 nm. MWC was determined using the gravimetric method; sediment particle size was measured using a laser particle size analyzer (LS13320, Beckman Coulter, Brea, CA, USA), and particle-size characteristics were represented by D50.

### 2.3. DNA Extraction, PCR Amplification, and High-Throughput Sequencing

For each sediment sample, three freeze-dried subsamples were used for genomic DNA extraction. Genomic DNA was extracted using the TGuide S96 Magnetic Bead Soil Genomic DNA Extraction Kit (DP812, Tiangen Biotechnology, Beijing, China), and the concentration of the DNA extracts was measured using a microplate reader (Synergy HTX, Gene Company Limited, Hong Kong, China) with 1× dsDNA HS Working Solution.

The V4 region of the 18S rRNA gene was amplified using the universal primer pair TAReuk454FWD1 (5′-CCAGCA(G/C)C(C/T)GCGGTAATTCC-3′) and TAReukREV3 (5′-ACTTTCGTTCTTGAT(C/T)(A/G)A-3′). Based on the DNA concentration and target amplification region, PCR amplification and detection were performed using the Belling 1000 Automated System (Ruifudi Biomedical Co., Ltd., Shanghai, China) to detect eukaryotic microorganisms in the samples. Equal volumes of the three PCR products from each sample were pooled. The pooled PCR products were verified by electrophoresis on a 1.8% agarose gel and subsequently purified [21].

Finally, high-throughput sequencing of the purified PCR products was performed on the Illumina NovaSeq 6000 platform. Quality-controlled sequences were denoised using DADA2 in QIIME 2 (version 2020.6) [28], and low-abundance ASVs were filtered using a threshold of 0.005% of the total sequence count. Taxonomic annotation of feature sequences was performed using a naïve Bayes classifier against the SILVA database [29]. The detailed procedures for DNA extraction, PCR amplification, and sequencing data processing were similar to those described in our previous study [23]. The raw sequence data were deposited in the NCBI Sequence Read Archive (SRA) under accession number PRJNA1456982.

### 2.4. Eukaryotic Microbial Community Structure and Biodiversity

Alpha-diversity indices, including Chao1, Shannon, and Simpson indices, were calculated using QIIME 2 to characterize the richness and diversity of sedimentary eukaryotic microbial communities [21]. A beta-diversity matrix was constructed based on Bray–Curtis dissimilarities at the ASV level, and principal coordinate analysis (PCoA) was used to visualize differences in community composition among sedimentary zones [30]. Permutational multivariate analysis of variance (PERMANOVA) was used to test the significance of differences in community composition among sedimentary zones [31].

Differences in the relative abundance of dominant taxa and alpha-diversity indices among sedimentary zones were analyzed using IBM SPSS Statistics 25. Depending on data distribution and homogeneity of variance, either one-way analysis of variance (ANOVA) or the Kruskal–Wallis test was used for among-group comparisons [32]. Linear discriminant analysis effect size (LEfSe) was used to identify taxa that discriminated among sedimentary zones, with the linear discriminant analysis (LDA) score threshold set to 3.5 [33]; analyses were performed on the Biomarker platform.

### 2.5. Analysis of Community-Environmental Factor Relationships

To elucidate the relationships between sedimentary eukaryotic microbial community differentiation and environmental factors, Spearman’s rank correlation coefficients between dominant orders and environmental variables were first calculated, and correlation bubble plots were generated. Subsequently, redundancy analysis (RDA) was conducted at the ASV level to examine the relationships between community composition and major environmental variables. RDA was performed using the ggvegan package in R.

To further evaluate the explanatory power of environmental factors for community variation, permutation tests and variance partitioning analysis (VPA) were performed using the vegan package in R. At the overall study-area scale, ordination analysis was conducted using NH_4_^+^-N, NO_3_^−^-N, TN, TP, TOC, MWC, D50, and pH to test the contribution of each environmental variable to community composition. At the sub-lake scale, separate RDA and VPA analyses were conducted for the four sedimentary zones—DP, GH, HX, and XH—to identify the primary environmental factors associated with each zone and their relative explanatory power. All analyses were based on environmental variable matrices corresponding to the sediment samples.

### 2.6. Functional Annotation of Fungi and Their Relationships with Environmental Factors

Because FunGuild is primarily designed to annotate the trophic modes and ecological guilds of fungal taxa, functional group analysis was further performed on annotatable fungal taxa in the ASV table [34]. First, the ASV taxonomic classification table was formatted according to the requirements of the FunGuild script; the taxonomy column was retained, and entries without annotation information were removed. Subsequently, the FunGuild v1.1 script was used to annotate identifiable fungal ASVs, generating both matched and unmatched annotation results.

Based on the annotation results, the trophic composition and relative abundance of annotated fungal groups were summarized across samples. Major functional groups, including pathotrophs, saprotrophs, and symbiotrophs, were identified, and their distributions across sedimentary zones and depth layers were compared. ASVs that could not be assigned to a functional type were labeled as Unassigned for subsequent analysis.

To further evaluate the relationship between the overall composition of annotated fungal functional groups and environmental gradients, a Mantel test was conducted based on the sample-by-trophic-mode matrix. First, the FunGuild annotation results were aggregated by sample to generate a trophic-mode relative abundance matrix, which was subjected to Hellinger transformation. The environmental variable matrix, including NH_4_^+^-N, NO_3_^−^-N, TN, TP, TOC, MWC, D50, and pH, was standardized using Z-score normalization. Subsequently, Bray–Curtis dissimilarities and Euclidean distances were calculated from the functional group matrix and environmental matrix, respectively, and the Mantel test was used to assess the association between variation in annotated fungal trophic-mode composition and environmental dissimilarity. Additionally, Spearman’s rank correlation coefficients were calculated among environmental factors to identify covariation patterns among environmental variables. Correlation analyses and visualization were performed in R (version 4.2.3).

### 2.7. Microbial Community Assembly

To quantitatively assess the relative contributions of deterministic and stochastic processes to the assembly of sedimentary eukaryotic microbial communities, the phylogeny-based null-model framework iCAMP was employed [35]. First, a phylogenetic tree was constructed based on ASV sequences, and taxonomic units were grouped into phylogenetic bins. Subsequently, the β-net correlation index (βNRI) and modified Raup–Crick index (RC) were calculated for each bin using the iCAMP package in R to infer the relative contributions of different ecological processes.

The classification criteria were as follows: βNRI < −1.96 indicated homogeneous selection (HoS), whereas βNRI > +1.96 indicated heterogeneous selection (HeS). For pairwise comparisons with |βNRI| ≤ 1.96, further classification was performed using RC: RC < −0.95 indicated homogenizing dispersal (HD), RC > +0.95 indicated dispersal limitation (DL), and |RC| ≤ 0.95 indicated undominated processes, mainly reflecting stochastic processes such as ecological drift (DR).

### 2.8. Co-Occurrence Network Construction and Analysis

To compare potential biological association patterns among eukaryotic microbial communities across sedimentary zones, co-occurrence networks were constructed for each zone using the ggClusterNet package in R, and network topological properties were compared using the meconetcomp package in R [22,36]. To reduce noise caused by low-abundance ASVs, ASVs with relative abundances below 0.5% were removed before network analysis. Subsequently, pairwise associations among ASVs were calculated using SparCC, and network edges were retained when |r| > 0.2 and the false discovery rate (FDR)-corrected *p*-value was <0.05.

Based on the constructed networks, topological metrics were calculated, including average degree (Average.Degree), average weighted degree (Avg.Weighted.Degree), average clustering coefficient (Avg.Clustering.Coefficient), average path length (Avg.Path.length), network diameter (Network.Diameter), and graph density (Graph.Density). ASV nodes were further classified according to their intra-module connectivity (Zi) and inter-module connectivity (Pi) as follows: peripheral nodes (Zi ≤ 2.5, Pi ≤ 0.62), module hubs (Zi > 2.5, Pi ≤ 0.62), connectors (Zi ≤ 2.5, Pi > 0.62), and network hubs (Zi > 2.5, Pi > 0.62). Nodes other than peripheral nodes were defined as potential keystone taxa. Network visualization was performed using Gephi (version 0.10.1).

## 3. Results

### 3.1. Physicochemical Properties of Lake Taihu Sediments

**Figure 2:**
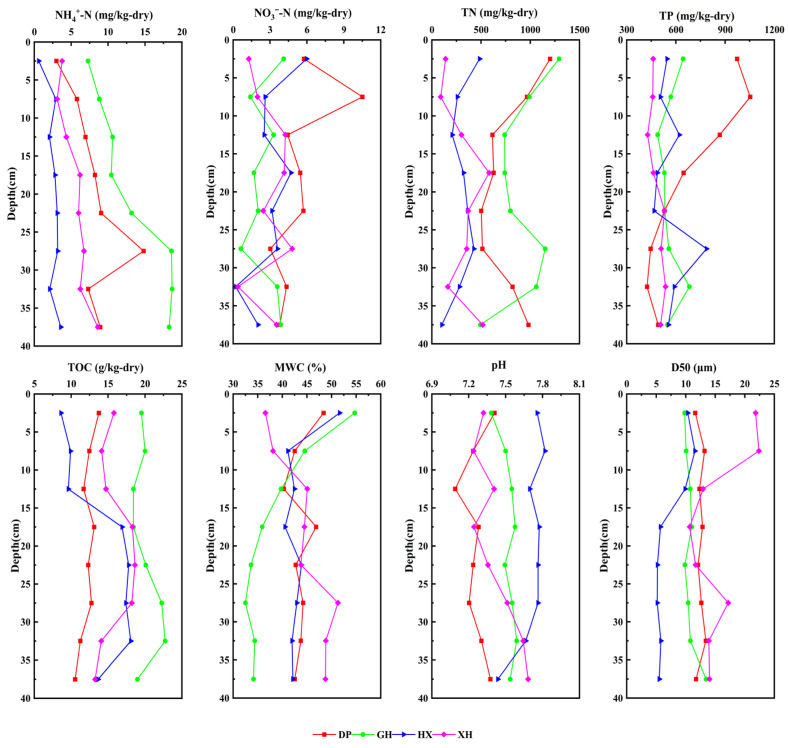
Physicochemical characteristics of sediments in different water areas of Lake Taihu. DP, Dapu; GH, Gonghu; HX, central Lake Taihu; XH, Xuhu. NH_4_^+^-N, ammonium nitrogen; NO_3_^−^-N, nitrate nitrogen; TN, total nitrogen; TP, total phosphorus; TOC, total organic carbon; MWC, moisture content; D50, median sediment particle size.

The physicochemical properties of sediments varied markedly among different zones of Lake Taihu as indicated by both the measured values and statistical comparisons (Figure 2, Tables S2 and S3). Based on the vertical distributions and inter-zone comparisons, nitrogen accumulation was particularly pronounced in the DP and GH zones. NH_4_^+^-N was highest in GH [11.90 (8.32)] and remained relatively high in DP [7.77 (2.31)], whereas lower values were observed in HX [2.87 (1.04)] and XH [6.11 (2.17)]. TN showed a similar pattern, with higher concentrations in GH (908.88 ± 261.31) and DP (779.01 ± 254.38) than in HX (306.44 ± 122.26) and XH (315.12 ± 177.76). Although TP was numerically highest in DP [588.85 (413.24)], its inter-zone difference was not statistically significant (*p* = 0.107). In contrast, TN and NH_4_^+^-N were generally lower in HX and XH, and the pairwise comparisons confirmed that TN in HX and XH was significantly lower than that in DP and GH, while NH_4_^+^-N in HX was significantly lower than that in DP and GH. These variables still varied across depth layers, suggesting vertical heterogeneity within the sediments.

Regarding sediment physical properties, D50 differed significantly among lake zones (*p* < 0.001), whereas MWC showed only a non-significant inter-zone trend (*p* = 0.096). The HX zone generally had smaller particle sizes, with a median D50 of 5.76 (4.57), indicating fine-grained sediment characteristics; in contrast, the XH zone had the largest median D50 [13.97 (5.73)], suggesting stronger hydrodynamic sorting and sediment transport effects in this area. MWC fluctuated with depth in all zones; its median value was lowest in GH [35.15 (6.95)] and relatively higher in DP [43.25 (2.35)], HX [42.31 (1.43)], and XH [44.79 (6.42)], although the overall inter-zone difference was not significant. pH also varied significantly among lake zones (*p* < 0.001), with the highest median value in HX [7.76 (0.07)] and the lowest in DP [7.26 (0.09)].

Statistical analysis showed that differences in sediment physicochemical properties were much more strongly associated with lake zones than with depth levels (lake zones: R^2^ = 0.554, *p* = 0.001; depth: R^2^ = 0.036, *p* = 0.887). Further comparisons indicated that NH_4_^+^-N, NO_3_^−^-N, TN, TOC, D50, and pH differed significantly among lake zones (*p* < 0.05), whereas TP and MWC did not show significant inter-zone differences (Table S3). Overall, variations in nutrient status, particle-size distribution, organic matter content, and pH among sedimentary zones provided an important environmental context for the differentiation of sedimentary eukaryotic microbial communities in Lake Taihu. These differences were likely related to the contrasting depositional settings of the four zones, including stronger riverine and agricultural inputs in DP, urban-related nutrient and organic matter inputs in GH, fine-grained sediment accumulation and open-lake hydrodynamic disturbance in HX, and hydrodynamic sorting or sediment transport effects in XH.

### 3.2. Analysis of Sedimentary Eukaryotic Microbial Community Structure and Spatial Variation

A total of 1725 ASVs were identified from the 96 samples; after screening, 568 ASVs were retained for subsequent analysis of eukaryotic microbial communities. The sequencing coverage of each sample approached 1.000, and the rarefaction curves tended to plateau, indicating that the sequencing depth was sufficient to capture the major eukaryotic microbial groups in the samples (Figure S1).

**Figure 3:**
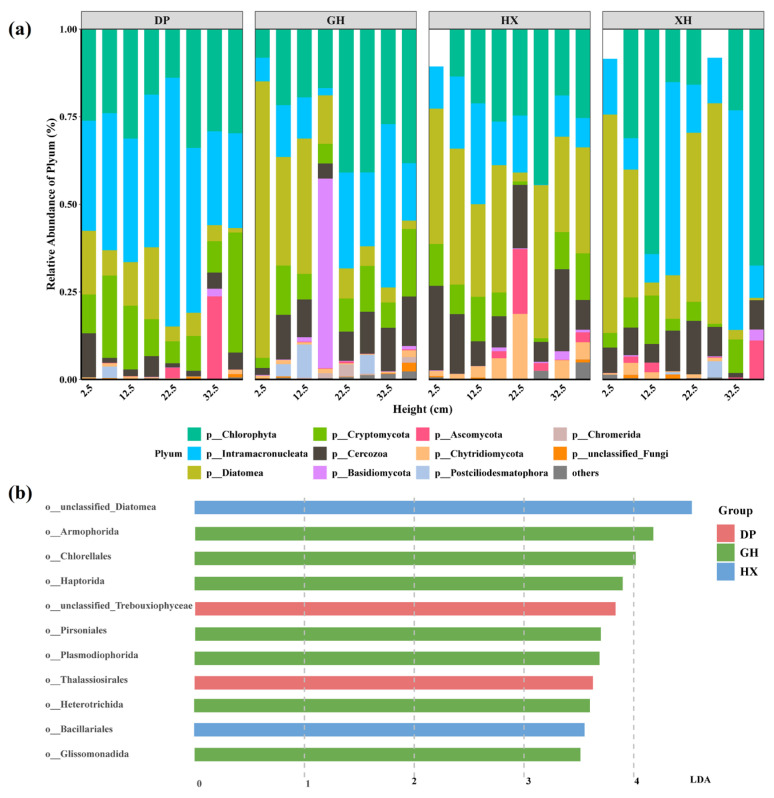
Composition of eukaryotic microbial communities in sediment layers of different research water areas in Lake Taihu. (**a**) Relative abundance of dominant eukaryotic microorganisms at phylum level in sediments from different sedimentary areas. (**b**) LDA bar chart. Indicator eukaryotic microorganisms with LDA score threshold of 3.5 in eukaryotic microbial communities related to the four sedimentary areas at order level. DP, Dapu; GH, Gonghu; HX, central Lake Taihu; XH, Xuhu; LDA, linear discriminant analysis. The prefixes “p__” and “o__” indicate phylum and order levels, respectively.

Taxonomic annotation showed that eukaryotic microbial communities in the four sedimentary zones of Lake Taihu comprised 16 phyla, 37 classes, 79 orders, 115 families, and 149 genera. At the dominant high-taxonomic level, the main groups across the study area were Chlorophyta (25.97%), Intramacronucleata (22.90%), and Diatomea (22.15%) (Figure 3a). The composition of dominant groups differed among lake zones: ciliates were relatively dominant in the DP zone; Chlorophyta accounted for a higher proportion in the GH zone; Diatomea remained relatively stable in the HX zone; and despite pronounced fluctuations among depth layers in the XH zone, Diatomea, Chlorophyta, and Intramacronucleata remained the main components overall. These differences may reflect zone-specific environmental filtering associated with nutrient status, organic matter, sediment texture, and hydrodynamic conditions.

Tests of between-group differences in dominant taxa revealed significant variation in Chromerida, Cercozoa, Intramacronucleata, and Chytridiomycota among lake zones. LEfSe analysis further identified differentially abundant taxonomic units across sedimentary zones (Figure 3b). Among the four zones, GH showed the highest number of enriched groups, including Cercozoa, Postciliodesmatophora, Cryptomycota, Intramacronucleata, and Chlorophyta, indicating a relatively complex community composition in this area, possibly related to its higher organic matter content and urban-influenced depositional environment. The DP zone was mainly enriched in unclassified_Trebouxiophyceae and Thalassiosirales, whereas the HX zone was characterized by unclassified_Diatomea and Bacillariales. Under the current threshold, no significantly enriched indicator taxa were detected in the XH zone. These results indicate that eukaryotic microbial communities in Lake Taihu sediments differed not only in overall composition but also in zone-specific distribution patterns of dominant taxa.

### 3.3. Analysis of Diversity Characteristics of Eukaryotic Microorganisms in Lake Taihu Sediments

**Figure 4:**
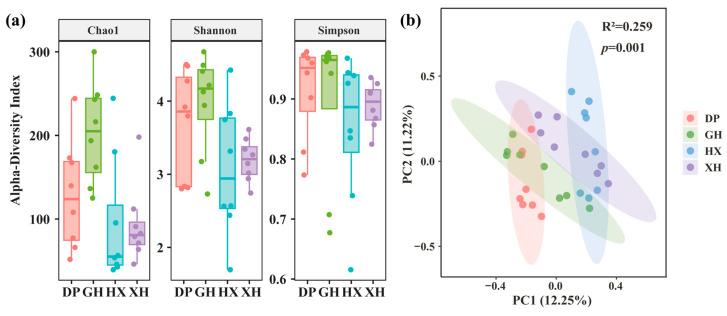
Microbial diversity characteristics and spatial heterogeneity patterns in Lake Taihu sediments (**a**) Microbial α-diversity; (**b**) Microbial β-diversity (horizontal axis is the first principal coordinate component, which can explain 12.25% of microbial community structure differences; vertical axis is the second principal coordinate component, which can explain 11.22% of microbial community structure differences). DP, Dapu; GH, Gonghu; HX, central Lake Taihu; XH, Xuhu; PC1 and PC2, the first and second principal coordinate axes.

Alpha-diversity analysis showed significant differences in eukaryotic microbial community diversity among lake zones (Figure 4a). Overall, the Chao1 and Shannon indices were generally higher in the GH zone, suggesting relatively high community richness and diversity in this area. In contrast, all three indices were generally lower in the HX zone, indicating lower community richness and evenness, whereas the DP and XH zones showed intermediate values. Collectively, these indices indicated that alpha diversity of eukaryotic microbial communities was generally higher in the GH zone and lower in the HX zone.

Patterns of vertical variation differed among lake zones (Table S4). In the DP and HX zones, the Chao1, Shannon, and Simpson indices generally decreased with depth, with a slight rebound in the bottom layer. In contrast, the GH and XH zones showed more pronounced depth-related changes in the Chao1 index, whereas changes in the Shannon and Simpson indices were relatively weak. This indicates that vertical variation in some zones was mainly reflected in species richness, while changes in evenness were relatively limited.

Beta-diversity analysis further revealed significant differentiation in community composition among sedimentary zones (Figure 4b). PCoA results showed that samples from the four lake zones displayed a clear separation trend in the ordination space, with samples from the HX zone more clearly separated from those of the other zones. PERMANOVA confirmed significant differences in community composition among lake zones (R^2^ = 0.259, *p* = 0.001), and all pairwise comparisons were significant (*p* = 0.001). These results suggest that eukaryotic microbial communities in Lake Taihu sediments exhibited significant spatial heterogeneity at the regional scale.

### 3.4. Environmental Drivers of Microbial Community Structure Differentiation

**Figure 5:**
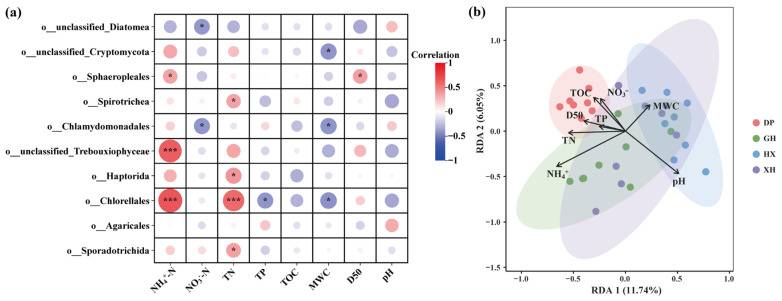
Relationships between dominant sedimentary eukaryotic orders and environmental factors in Lake Taihu. (**a**) Spearman correlations between the top 10 dominant eukaryotic orders and sediment environmental factors. Circle size indicates correlation strength; red and blue indicate positive and negative correlations, respectively. Asterisks indicate significance levels (* *p* < 0.05, *** *p* < 0.001). (**b**) Redundancy analysis (RDA) showing the relationships between ASV-level eukaryotic community composition and environmental variables across the four sediment zones. DP, Dapu; GH, Gonghu; HX, central Lake Taihu; XH, Xuhu. NH_4_^+^-N, ammonium nitrogen; NO_3_^−^-N, nitrate nitrogen; TN, total nitrogen; TP, total phosphorus; TOC, total organic carbon; MWC, moisture content; D50, median sediment particle size. The prefixes “o__” indicate order levels.

Spearman’s correlation analysis between dominant orders and environmental factors showed that sediment nutrient levels and physical properties were widely associated with eukaryotic microbial community composition (Figure 5a). Specifically, NH_4_^+^-N was significantly positively correlated with Chlorellales and unclassified_Trebouxiophyceae and was also positively correlated with Sphaeropleales; NO_3_^−^-N was significantly negatively correlated with unclassified_Diatomea and Chlamydomonadales; and TN showed positive correlations of varying strength with Chlorellales, Spirotrichea, Haptorida, and Sporadotrichida. Furthermore, D50, MWC, TP, and TOC were also significantly correlated with several dominant taxa, indicating that nutrient availability, particle-size structure, and moisture conditions in the sedimentary environment jointly contributed to community distribution patterns.

RDA further revealed the overall relationships between community composition and environmental factors across lake zones (Figure 5b). At the whole-study-area scale, the first two RDA axes jointly explained 17.79% of the community variation, with RDA1 and RDA2 explaining 11.74% and 6.05%, respectively. Samples from different lake zones showed partial separation in the ordination space: DP was mainly associated with the directions of TOC, D50, TP, and TN; GH was closer to the NH_4_^+^-N vector; HX was distributed mainly along the pH, NO_3_^−^-N, and MWC directions; and XH was associated with multiple environmental factors. The 999-permutation test showed that only NH_4_^+^-N was significant (F = 3.2057, *p* = 0.001), whereas D50 showed marginal significance (F = 1.5673, *p* = 0.082); the remaining environmental factors were not significant (*p* > 0.05). VPA showed that NH_4_^+^-N, TP, D50, TOC, and NO_3_^−^-N explained 6.45%, 1.76%, 0.91%, 0.85%, and 0.33% of the variation in community composition, respectively. NH_4_^+^-N had the highest explanatory contribution, suggesting that it was the primary measured environmental factor associated with the spatial differentiation of eukaryotic microbial communities across Lake Taihu. Furthermore, the model residual variance was 0.267453, suggesting that sedimentary eukaryotic microbial community differentiation may also be influenced by unmeasured environmental variables, biological interactions, and stochastic processes beyond the measured physicochemical factors.

RDA conducted for each lake subregion further revealed clear regional differences in environmental drivers. In DP, TP was the only significant environmental factor (F = 1.6861, *p* = 0.046). In GH, both NH_4_^+^-N and TN were significant, with NH_4_^+^-N showing the strongest explanatory power (NH_4_^+^-N: F = 8.4296, *p* = 0.001; TN: F = 2.8630, *p* = 0.003). In HX, NH_4_^+^-N was significant (F = 2.0123, *p* = 0.022). In XH, both MWC and TOC were significant (MWC: F = 3.4702, *p* = 0.001; TOC: F = 3.8803, *p* = 0.002).

Overall, the environmental drivers of eukaryotic microbial communities in Lake Taihu sediments showed clear scale dependence and regional specificity. At the whole-study-area scale, NH_4_^+^-N was the primary explanatory factor. At the sub-lake scale, DP was mainly associated with TP, GH with NH_4_^+^-N and TN, HX with NH_4_^+^-N, and XH with the combined effects of TOC and MWC.

### 3.5. Spatial Distribution Characteristics and Influencing Factors of Annotated Fungal Functional Groups

**Figure 6:**
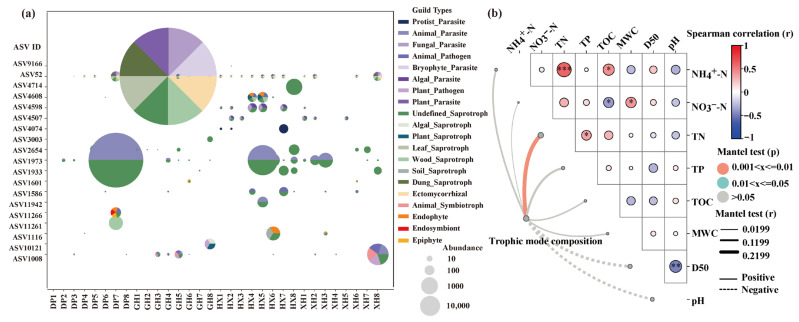
Distribution of annotatable fungal functional groups in different sedimentary zones of Lake Taihu and their correlations with environmental factors. (**a**) Functional-group composition and relative abundance of major annotatable fungal ASVs across samples. Bubble size indicates ASV relative abundance, and sector color denotes functional-group type. (**b**) Mantel correlations between trophic mode composition and environmental factors, and Spearman correlations among environmental variables. Line width represents Mantel’s r, line type indicates positive or negative correlations, and line color denotes significance. In the upper triangular bubble plot, bubble color indicates the direction and strength of Spearman correlations, and asterisks indicate significance levels (* *p* < 0.05; ** *p* < 0.01; *** *p* < 0.001). DP, Dapu; GH, Gonghu; HX, central Lake Taihu; XH, Xuhu. NH_4_^+^-N, ammonium nitrogen; NO_3_^−^-N, nitrate nitrogen; TN, total nitrogen; TP, total phosphorus; TOC, total organic carbon; MWC, moisture content; D50, median sediment particle size.

FunGuild functional annotation showed that annotated fungal groups in different sedimentary zones of Lake Taihu primarily comprised pathotrophs, saprotrophs, symbiotrophs, and their composite trophic modes (Figure 6, Tables S5–S8), with marked differences across lake zones and depth layers. Overall, functional groups in some samples were dominated by a few high-abundance ASVs and showed clear regional clustering.

In the DP zone, ASV1973, assigned to the genus *Saccharomycopsis* and the Pathotroph-Saprotroph mode, was dominant, accounting for 81.14% of the total abundance, and was mainly enriched in the 30–35 cm layer. The GH zone showed the highest dominance of a single functional ASV, with ASV52, assigned to the order Agaricales and the Pathotroph-Saprotroph-Symbiotroph mode, accounting for 96.39% of the total abundance and mainly distributed in the 15–20 cm layer. In the HX zone, functional groups were more evenly distributed; although ASV1973 remained dominant, its relative abundance decreased to 42.81%, and several moderately abundant groups were also present. In the XH zone, ASV1008, assigned to the genus *Pichia*, and ASV1973 were co-dominant, accounting for 43.30% and 29.60% of the total abundance, respectively. These ASVs were enriched in the surface and deep layers but were less represented in the middle layer.

The response patterns of dominant functional groups to environmental factors varied across lake zones (Figures S2–S5). No significant correlations were detected in the DP zone. In the GH zone, ASV52 was significantly negatively correlated with both TOC and TP. In the HX zone, ASV1973 was significantly positively correlated with NH_4_^+^-N and TOC and significantly negatively correlated with D50. In addition, ASV52 was significantly positively correlated with TN, ASV4598, assigned to the family Chytridiaceae, was significantly negatively correlated with TP, and ASV1933 was significantly negatively correlated with NO_3_^−^-N. In the XH zone, ASV1973 was significantly negatively correlated with TP, ASV52 was significantly positively correlated with D50, ASV2654, assigned to the genus *Saccharomyces*, was significantly positively correlated with MWC, and ASV4507, assigned to the family Chytridiaceae, was significantly negatively correlated with MWC. These results indicate that annotated fungal functional groups showed zone-specific responses to nutrient status, organic carbon, particle size, and moisture conditions.

To further assess the influence of environmental gradients on overall functional-group structure, a Mantel test was conducted based on the sample-by-trophic-mode matrix (Figure 6b). Among all environmental factors, only TN was significantly positively correlated with differences in annotated fungal trophic-mode composition (Mantel’s r = 0.277, *p* = 0.002), whereas NH_4_^+^-N, NO_3_^−^-N, TP, TOC, MWC, D50, and pH were not significantly correlated with trophic-mode composition. Spearman’s correlation analysis further showed that NH_4_^+^-N was significantly positively correlated with TN and TOC; NO_3_^−^-N was significantly negatively correlated with TOC and significantly positively correlated with MWC; TN was significantly positively correlated with TP; and D50 was significantly negatively correlated with pH, suggesting covariation among sedimentary environmental variables. The Mantel results indicate that differentiation in annotated fungal trophic modes in Lake Taihu sediments was associated with environmental heterogeneity, but at the univariate level, this association was mainly reflected in the significant response to TN.

Overall, fungal functional groups in different sedimentary zones of Lake Taihu showed distinct regional differences in dominant ASV composition, trophic structure, and environmental association patterns. Functional-group structures in the DP and GH zones were relatively concentrated, whereas those in the HX zone were more dispersed; the XH zone was characterized by the co-dominance of two dominant ASVs. These patterns indicate that sedimentary environmental heterogeneity was closely associated with the spatial distribution of fungal functional groups.

### 3.6. Mechanisms of Microbial Community Assembly

**Figure 7:**
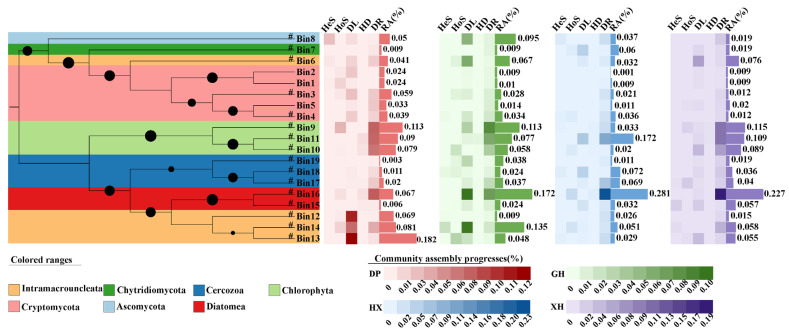
Microbial community construction mechanisms. Left side shows phylogenetic tree of Bin composition, with branches containing high-abundance Bins marked #, and background color of phylogenetic tree set according to phylum classification information. The size of the black dots is proportional to the relative abundance of the corresponding Bin. Right side shows heatmaps of various indicator data for assembly processes in DP, GH, HX, and XH, respectively, and bar charts of abundance data for each Bin. DP, Dapu; GH, Gonghu; HX, central Lake Taihu; XH, Xuhu; Hes, heterogeneous selection; HoS, homogeneous selection; DL, dispersal limitation; HD, homogenizing dispersal; DR, drift; RA, relative abundance.

iCAMP-based community assembly analysis indicated that eukaryotic microbial community assembly in the four sedimentary zones of Lake Taihu was generally dominated by stochastic processes (Figure 7). Dispersal limitation (DL), drift (DR), and homogeneous selection (HoS) were the primary assembly processes, but their relative contributions varied among lake zones. In the GH zone, dispersal limitation was the dominant process, followed by drift. In the DP, HX, and XH zones, drift was dominant, followed by dispersal limitation, whereas the contributions of heterogeneous selection (HeS) and homogenizing dispersal (HD) were generally low.

Further analysis of ecological-process contributions at the bin level showed that the 568 ASVs were classified into 19 phylogenetic bins, mainly including Chlorophyta, Diatomea, Intramacronucleata, Cercozoa, Cryptomycota, and Ascomycota. In the GH zone, bins with higher contributions from dispersal limitation were mainly concentrated in phylogenetic branches related to Diatomea and Intramacronucleata. In contrast, in the DP, HX, and XH zones, bins with higher drift contributions mainly originated from branches related to Chlorophyta and Diatomea. These results indicate that although stochastic processes dominated the assembly of eukaryotic microbial communities in Lake Taihu sediments, the dominant stochastic processes differed among sedimentary zones.

### 3.7. Co-Occurrence Network Characteristics of Eukaryotic Microorganisms

**Figure 8:**
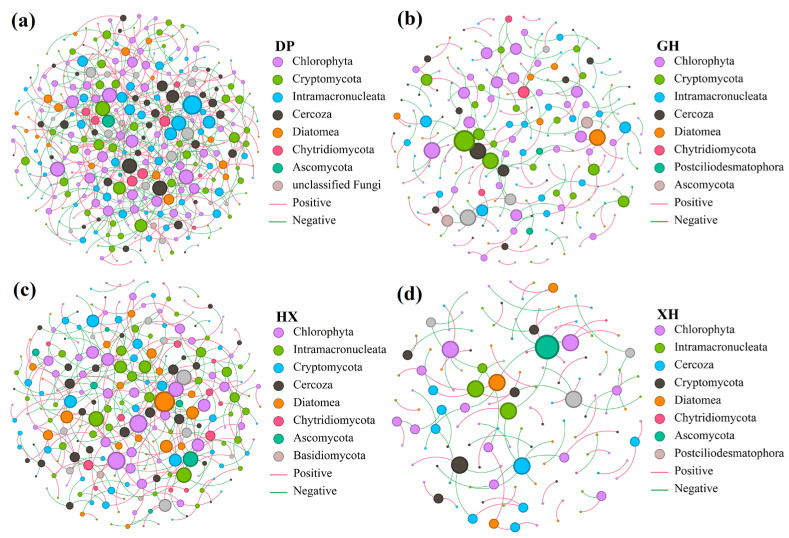
Microbial co-occurrence networks. (**a**) Co-occurrence network of eukaryotic microbial communities in DP; (**b**) co-occurrence network of eukaryotic microbial communities in GH; (**c**) co-occurrence network of eukaryotic microbial communities in HX; (**d**) co-occurrence network of eukaryotic microbial communities in XH. The networks were constructed based on SparCC correlation analysis. Nodes in co-occurrence networks of different sedimentary areas are color-coded according to microbial phylum, and edges are color-distinguished according to positive/negative correlations. DP, Dapu; GH, Gonghu; HX, central Lake Taihu; XH, Xuhu. Nodes represent ASVs, and node colors indicate major taxonomic groups. Edges represent potential associations between ASVs, with red and green lines indicating positive and negative associations, respectively.

SparCC-based co-occurrence networks of eukaryotic microbial communities revealed marked differences in potential biological association patterns among sedimentary zones (Figure 8). In terms of network size, the GH zone had the largest number of nodes (396), the DP zone had the largest number of edges (1878), and the XH zone had the fewest edges (903). In terms of connectivity metrics, the DP and HX zones had higher average degree values (11.313 and 9.308), average weighted degree values (2.798 and 2.280), and network densities (0.034 and 0.036) than the GH and XH zones, indicating that the networks in DP and HX were more compact, with tighter connections among nodes. In addition, the DP and HX zones had shorter average path lengths (2.662 and 2.746) and smaller network diameters (4 and 5), whereas the GH and XH zones had longer average path lengths (3.319 and 3.503) and larger network diameters (6 and 7), suggesting that the latter two networks were more dispersed. Modular-structure analysis showed that the GH and XH zones each contained 12 modules, more than the DP and HX zones, which each contained 10 modules. The XH and GH zones also showed higher modularity values (0.419 and 0.373), indicating more pronounced modular differentiation. In contrast, the DP and HX zones had lower modularity values (0.294 and 0.324), indicating closer connections among modules. Overall, the DP and HX zones exhibited compact network structures characterized by high connectivity and density, whereas the GH and XH zones exhibited more dispersed network structures characterized by higher modularity and lower connectivity. This pattern reflects clear regional differentiation in potential biological associations among eukaryotic microbial communities across sedimentary zones of Lake Taihu. Detailed network parameters are shown in Table S9.

Gephi-based modular analysis and assessment of potential keystone taxa showed that connector nodes were the most abundant node type in the co-occurrence networks of all four sedimentary zones (Figure S6). Among these zones, DP, GH, and HX contained network hub nodes, and their modules exhibited both functional differentiation and integration. The DP zone contained one network hub node, which belonged to the phylum Cercozoa and the class Thecofilosea. This network included five primary-production modules represented by Diatomea and Chlorophyta, three fungal-decomposition modules represented by Cryptomycota and Ascomycota, and two protozoan modules represented by Intramacronucleata and Cercozoa. The network hub nodes in both the GH and HX zones belonged to the phylum Chlorophyta and the class Chlorophyceae. The GH zone showed a distributed core structure, with two network hub nodes belonging to different modules, suggesting stronger functional integration. In contrast, the HX zone had a single green algal network core, indicating a high degree of centralization of primary-production functions. In the XH zone, the absence of network hub nodes suggested a relatively decentralized network-regulation pattern. Diatoms and Chlorophyta, as primary producers, were widely distributed across multiple modules, whereas protozoan groups, including Intramacronucleata and Cercozoa, and fungal groups, including Ascomycota and Cryptomycota, were relatively concentrated.

## 4. Discussion

### 4.1. Differences in Microbial Community Diversity and Environmental Drivers

Differentiation of eukaryotic microbial community structure in Lake Taihu sediments was closely associated with sediment nutrients, environmental conditions, and physical properties; regional differences in physicochemical properties may contribute to variations in microbial community diversity [37,38]. NH_4_^+^-N and NO_3_^−^-N, as major inorganic nitrogen forms in sediments, are important components of nitrogen cycling involving eukaryotic microorganisms [39]. In this study, NH_4_^+^-N concentrations in all four zones were significantly positively associated with green algal groups, including Chlorellales and unclassified_Trebouxiophyceae. This may be because NH_4_^+^-N can be directly assimilated during microbial growth and organic matter decomposition, serving as a preferred nitrogen source for eukaryotic microorganisms [40]. This pattern was particularly pronounced in the GH zone, consistent with the characteristics of ammonia nitrogen inputs from urban wastewater [41]. The negative associations between NO_3_^−^-N and diatoms, represented by unclassified_Diatomea, and Chlorophyta, represented by Chlamydomonadales, may be related to changes in sediment redox conditions and dissolved oxygen availability during nitrogen transformation. Previous studies have shown that nitrification can reduce sediment redox potential and maintain a more reducing environment [42], which may disrupt oxygen-dependent photosynthetic electron transport and respiratory metabolism in aerobic photosynthetic microorganisms and damage photosynthetic pigments and membrane structures [43]. In addition, excessively high rates of nitrification, denitrification, or anaerobic ammonium oxidation in sediments may accelerate inorganic nitrogen turnover and depletion, thereby reducing nitrogen availability for heterotrophic microorganisms [44,45]. In addition to eukaryotic photosynthetic microorganisms, cyanobacteria are ecologically important primary producers in eutrophic lake ecosystems and are closely associated with recurrent algal blooms in Lake Taihu. The deposition of cyanobacterial biomass and bloom-derived organic matter can alter sediment nutrient regeneration, organic carbon availability, and microhabitat conditions, thereby providing an important ecological background for interpreting the spatial differentiation of sedimentary eukaryotic microbial communities.

TP is associated with essential biochemical structures, including microbial ATP, RNA, DNA, and membrane phospholipids, and is involved in key life processes such as cell growth, division, energy metabolism, and signal transduction [46]. In the DP zone, the vertical gradient of TP, which decreased from the surface to deeper layers, may have contributed to community differentiation. This may be partly because high concentrations of inorganic phosphorus can inhibit ADP-glucose pyrophosphorylase, a key enzyme involved in carbohydrate synthesis, thereby constraining carbohydrate production [47]. Under phosphorus-limited conditions, sedimentary eukaryotic microbial communities may exhibit functional differentiation, favoring taxa with efficient phosphorus acquisition mechanisms [48].

In sedimentary environments, TOC provides an important carbon source for microbial growth and reproduction and influences ecological processes such as the degradation and transformation of organic micropollutants [49]. In low-TOC environments, sedimentary microbial biomass generally shows a positive relationship with TOC, whereas high TOC concentrations may promote the deposition of flocculated humic substances, thereby limiting microbial degradation and potentially constraining further increases in sedimentary microbial biomass [50,51].

MWC supports microbial life processes and may shape community diversity by altering sediment redox potential and substrate availability, thereby favoring eukaryotic microbial taxa adapted to different moisture conditions [52]. In the XH zone, MWC and TOC appeared to form a dual regulatory framework involving physical conditions and organic carbon availability. MWC may enhance the microbial availability of TOC by modifying the sediment microenvironment; together, these factors form a coupled physical–nutritional gradient that improves the explanation of community variation [53]. In the GH zone, MWC was significantly lower than that in the other sedimentary zones, and eukaryotic microbial biomass was significantly positively correlated with MWC. This may indicate that, under low-MWC conditions, moisture availability can become a limiting factor for microbial biomass and activity, and increased moisture content may support microbial biomass accumulation [54].

### 4.2. Equilibrium of Sedimentary Fungal Functional Groups Under Environmental Differentiation

This study showed that the diversity of annotated fungal functional groups in Lake Taihu sediments reflected a pattern of reciprocal regulation between environmental factors and community responses. In the DP and GH zones, the high relative abundance of strongly dominant taxa suggested that sedimentary environmental selection may substantially shape community function [55]. In the DP zone, the low-TOC and high-porosity sedimentary environment may have favored the pathotroph–saprotroph mixed-trophic genus Saccharomycopsis as the core functional group, whereas in the GH zone, the pathotroph–saprotroph–symbiotroph mixed-trophic order Agaricales dominated under low-phosphorus and low-TOC conditions. This result is consistent with the study by Keck et al. on lake sediments in France, suggesting that low-nutrient or polluted environments may promote functional-group homogenization and enhance the ecological contribution of core functional groups under specific resource-limiting conditions [56]. In addition, this study found that saprotrophic fungi and pathotroph–saprotroph composite groups may perform important ecological functions in lake sediments, including organic matter decomposition and nutrient cycling, reflecting efficient resource use and multifunctionality [57,58].

In contrast, the HX and XH zones showed more moderate dominance within fungal functional groups and greater diversity of functional categories. In the HX zone, ultrafine-grained sediments and a hypoxic microenvironment may have promoted the enrichment of Saccharomycopsis taxa, whereas TOC and nitrogen gradients supported the coexistence of other functional groups, resulting in functional diversification and complementarity within the community [59]. In contrast, the XH zone showed an alternating pattern of functional-group enrichment and suppression, with Pichia and Saccharomycopsis dominating at different depths, suggesting that environmental instability may promote dynamic ecological strategies that help maintain overall functionality [60]. Previous studies have shown that sediment vertical gradients and environmental heterogeneity can sustain high functional diversity and help communities maintain ecological homeostasis through interactions among functional groups [61].

Differences in functional diversity and ecological strategies also reflect community responses to nutrient gradients, organic carbon availability, sediment texture, and moisture conditions; sedimentary fungal functional groups may cope with environmental disturbances through network restructuring and functional redundancy, thereby maintaining ecosystem functions [62,63]. In the DP and GH zones, where resource constraints or physical stresses were more pronounced, communities appeared to concentrate functions within dominant groups to maintain stable core functions, reflecting a high-stability but low-diversity strategy. In the HX and XH zones, where environmental conditions were more complex or gradients varied more strongly, the coexistence of multiple groups and functional diversification may reduce ecological risk and enhance community stability.

### 4.3. Ecological Adaptation Mechanisms Driven by Community Assembly and Biological Interactions

Based on iCAMP and co-occurrence network analyses, this study found that eukaryotic microbial communities in Lake Taihu sediments exhibited a multilevel ecological adaptation pattern shaped by stochastic processes, environmental selection, and biological interactions. Regarding community assembly, stochastic processes dominated microbial community assembly in Lake Taihu sediments, which may be related to the physical characteristics of shallow lake ecosystems and the dispersal capacity of microbial communities [64,65]. Dispersal limitation played a dominant role in community assembly in the GH zone, possibly because weak hydrodynamic conditions in this zone prolonged water residence time and reduced microbial dispersal [66]. Ecological drift played a primary role in microbial community assembly in the DP, HX, and XH zones. This may be related to strong hydrodynamic exchange near river inflows in the DP zone and extensive stochastic dispersal driven by wind-induced circulation in the HX zone [67]. The XH zone, located near the Xukou Water Conservancy Hub in Suzhou, experiences frequent exchange with external water bodies, which may increase the influence of ecological drift on microbial communities [68].

Although stochastic processes dominated, deterministic processes still contributed to community structure. This study found that homogeneous selection contributed more to microbial community assembly than heterogeneous selection in all four sedimentary zones, suggesting that environmental filtering tended to promote compositional convergence among microbial communities during assembly [69]. This may be attributable to rapid lake-wide wind-driven mixing in shallow Lake Taihu, which can promote convergence in physicochemical conditions across samples [70]. Overall, our results indicate that both deterministic and stochastic processes played important roles in microbial community assembly.

At the level of biological interactions, patterns in the co-occurrence network structure of eukaryotic microorganisms in Lake Taihu sediments reflected a dynamic balance between environmental selection and biological interactions in freshwater sedimentary ecosystems. In the XH and GH zones, which receive relatively strong terrestrial inputs, high nutrient loads and anoxic sediment conditions may strongly filter microbial communities, leading to functional-group simplification and reduced network connectivity [71]. In contrast, the more interaction-dominated networks in the DP and HX zones were similar to microbial network patterns observed in relatively stable sedimentary environments; in these zones, more stable sedimentary conditions may allow interspecific interactions to play a stronger role in community organization [72]. The distinct ecological adaptation patterns observed across the four sedimentary zones of Lake Taihu reflect the adaptive strategies of freshwater sedimentary eukaryotic microbial communities under different hydrodynamic-dispersal regimes and sedimentary environmental constraints, including terrestrial inputs, nutrient enrichment, wind-induced mixing, water-exchange disturbance, and sediment habitat heterogeneity.

This study was based on total sedimentary DNA and 18S rRNA gene amplicon sequencing; therefore, the detected sequences may include DNA from active, dormant, or dead cells, as well as extracellular or partially degraded DNA preserved in sediments. Because DNA integrity analysis, viability treatment, RNA-based sequencing, qPCR, and fragment-size profiling were not performed, the proportion of degraded DNA could not be reliably estimated. Accordingly, the observed community patterns should be interpreted as the amplifiable sedimentary eukaryotic DNA pool rather than exclusively active communities. Future studies combining DNA- and RNA-based approaches or viability assays are needed to distinguish active microbial signals from residual sedimentary DNA.

## 5. Conclusions

This study compared the structure of eukaryotic microbial communities, environmental drivers, fungal functional groups, community assembly processes, and co-occurrence network patterns across four typical sedimentary zones in Lake Taihu. The results revealed significant spatial heterogeneity in eukaryotic microbial community structure and biodiversity across the four zones, with alpha diversity highest in the GH zone and lowest in the HX zone. Furthermore, vertical variation patterns differed among lake zones, and beta diversity showed significant differences among lake zones. Community differentiation was mainly associated with nutrients, including NH_4_^+^-N, NO_3_^−^-N, TP, and TN, and physicochemical properties, including median sediment particle size and moisture content, with these factors showing clear zone-specific characteristics. Community assembly was predominantly governed by stochastic processes, with dispersal limitation being the dominant process in the GH zone and ecological drift being dominant in the DP, HX, and XH zones. Annotated fungal functional groups differed significantly in trophic composition and spatial distribution among sedimentary zones, indicating that sedimentary environmental heterogeneity influenced their regional distribution patterns. Co-occurrence network patterns of sedimentary eukaryotic microorganisms were also closely associated with hydrodynamic-dispersal regimes and sedimentary environmental constraints. The XH and GH zones were characterized by higher modularity and lower connectivity, whereas the DP zone exhibited a highly connected, core-hub network organization.

These findings systematically reveal the structural characteristics, assembly mechanisms, and functional responses of eukaryotic microbial communities under different sedimentary environments in Lake Taihu. They broaden the understanding of eukaryotic microbial ecology in Lake Taihu sediments and provide a scientific basis for eutrophic lake ecosystem management and water-quality protection. In practical terms, the observed differentiation in sedimentary eukaryotic DNA profiles among depositional zones suggests that these molecular community patterns may serve as useful biological indicators for identifying sediment environmental heterogeneity and potential ecological stress. The zone-specific molecular community patterns identified in this study may support targeted sediment monitoring, pollution-risk assessment, and differentiated ecological restoration strategies in Lake Taihu and other large shallow eutrophic lakes.

## Data Availability

The original data presented in the study are openly available in the NCBI SRA database (No. PRJNA1456982).

## Supplementary Materials

The following supporting information can be downloaded at: https://www.mdpi.com/article/10.3390/microorganisms14051121/s1, Figure S1: Rarefaction curve of the sample; Figure S2: Correlation between functional eukaryotic microorganisms and environmental factors in the DP zone of Lake Taihu; Figure S3: Correlation between functional eukaryotic microorganisms and environmental factors in the GH zone of Lake Taihu; Figure S4: Correlation between functional eukaryotic microorganisms and environmental factors in the HX zone of Lake Taihu; Figure S5: Correlation between functional eukaryotic microorganisms and environmental factors in the XH zone of Lake Taihu; Figure S6: Zi-Pi plots for each network; Table S1: Sampling sites and their geographic coordinates; Table S2: Specific results of environmental factors; Table S3: Statistical comparison of sediment environmental variables among four depositional zones in Lake Taihu; Table S4: Vertical variation in alpha diversity indices of sedimentary eukaryotic microbial communities across different depositional zones of Lake Taihu; Table S5: FUNGuild functional annotation results of fungal ASVs from different sediment depths in the DP depositional zone of Lake Taihu; Table S6: FUNGuild functional annotation results of fungal ASVs from different sediment depths in the GH depositional zone of Lake Taihu; Table S7: FUNGuild functional annotation results of fungal ASVs from different sediment depths in the HX depositional zone of Lake Taihu; Table S8: FUNGuild functional annotation results of fungal ASVs from different sediment depths in the XH depositional zone of Lake Taihu; Table S9: Topological parameters of microbial co-occurrence networks in four depositional zones of Lake Taihu.
