# Supplementary material for: Spatial Patterns and Ecological Drivers of Sedimentary Eukaryotic Microorganisms Across Typical Depositional Zones of Lake Taihu
Source: Microorganisms. 2026 May 15;14(5):1121. doi: 10.3390/microorganisms14051121 (PMC13209375; doi:10.3390/microorganisms14051121)
Supplement: Supplementary file 1 [file microorganisms-14-01121-s001.zip › microorganisms-4319987-supplementary.pdf]

Spatial patterns and ecological drivers of sedimentary eukaryotic microorganisms across typical depositional zones of Lake Taihu

Zhendong Li [a], Yang Chen [a], Yajie Li [a], Aidong Ruan [a] *

[a] *College of Hydrology and Water Resources, Hohai University, Nanjing, 210098, China.**

Corresponding Authors:

Aidong Ruan, E-mail: adruan@hhu.edu.cn

CONTENTS:

Figure S1. Rarefaction curve of the sample.

Figure S2. Correlation between functional eukaryotic microorganisms and environmental factors in the DP zone of Lake Taihu.

Figure S3. Correlation between functional eukaryotic microorganisms and environmental factors in the GH zone of Lake Taihu (*, $P < 0.05$; **， $P < 0.01$; ***， $P < 0.001$).

Figure S4. Correlation between functional eukaryotic microorganisms and environmental factors in the HX zone of Lake Taihu (*, $P < 0.05$; **， $P < 0.01$; ***， $P < 0.001$).

Figure S5. Correlation between functional eukaryotic microorganisms and environmental factors in the XH zone of Lake Taihu (*, $P < 0.05$; **， $P < 0.01$; ***， $P < 0.001$).

Figure S6. Zi-Pi plots for each network.

Table S1. Sampling sites and their geographic coordinates.

Table S2. Specific results of environmental factors. Ammonia nitrogen, nitrate nitrogen, total nitrogen, total phosphorus, total organic carbon, mass water content, median particle diameter and pH were abbreviated as NH_4^+-N, NO_3^--N, TN, TP, TOC, MWC, D50 and pH, respectively.

Table S3. Statistical comparison of sediment environmental variables among four depositional zones in Lake Taihu.

Table S4. Vertical variation in alpha diversity indices of sedimentary eukaryotic microbial communities across different depositional zones of Lake Taihu.

Table S5. FUNGuild functional annotation results of fungal ASVs from different sediment depths in the DP depositional zone of Lake Taihu.

Table S6. FUNGuild functional annotation results of fungal ASVs from different sediment depths

in the GH depositional zone of Lake Taihu.

Table S7. FUNGuild functional annotation results of fungal ASVs from different sediment depths in the HX depositional zone of Lake Taihu.

Table S8. FUNGuild functional annotation results of fungal ASVs from different sediment depths in the XH depositional zone of Lake Taihu.

Table S9. Topological parameters of microbial co-occurrence networks in four depositional zones of Lake Taihu.

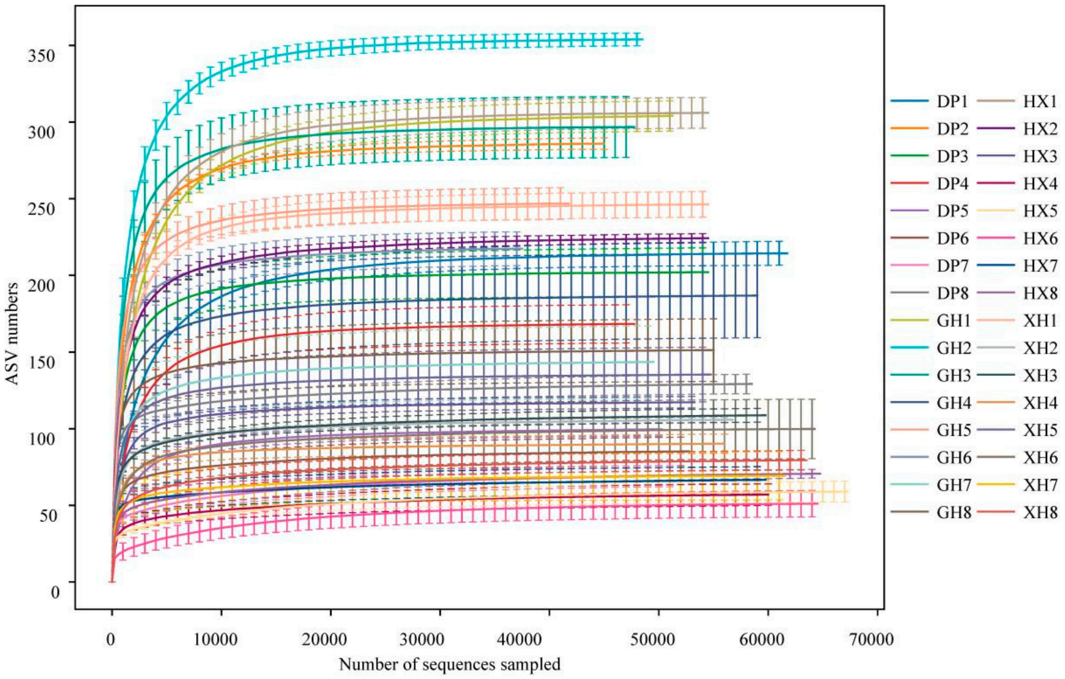

Figure S1. Rarefaction curve of the sample.

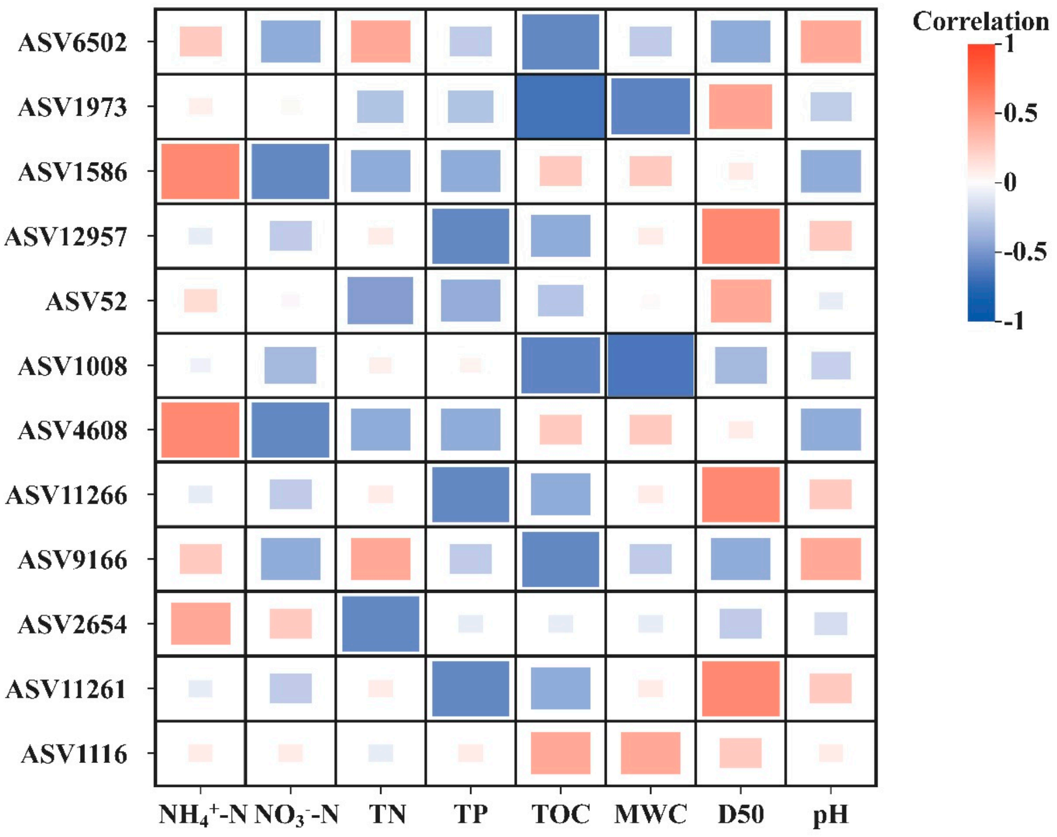

Figure S2. Correlation between functional eukaryotic microorganisms and environmental factors in the DP zone of Lake Taihu.

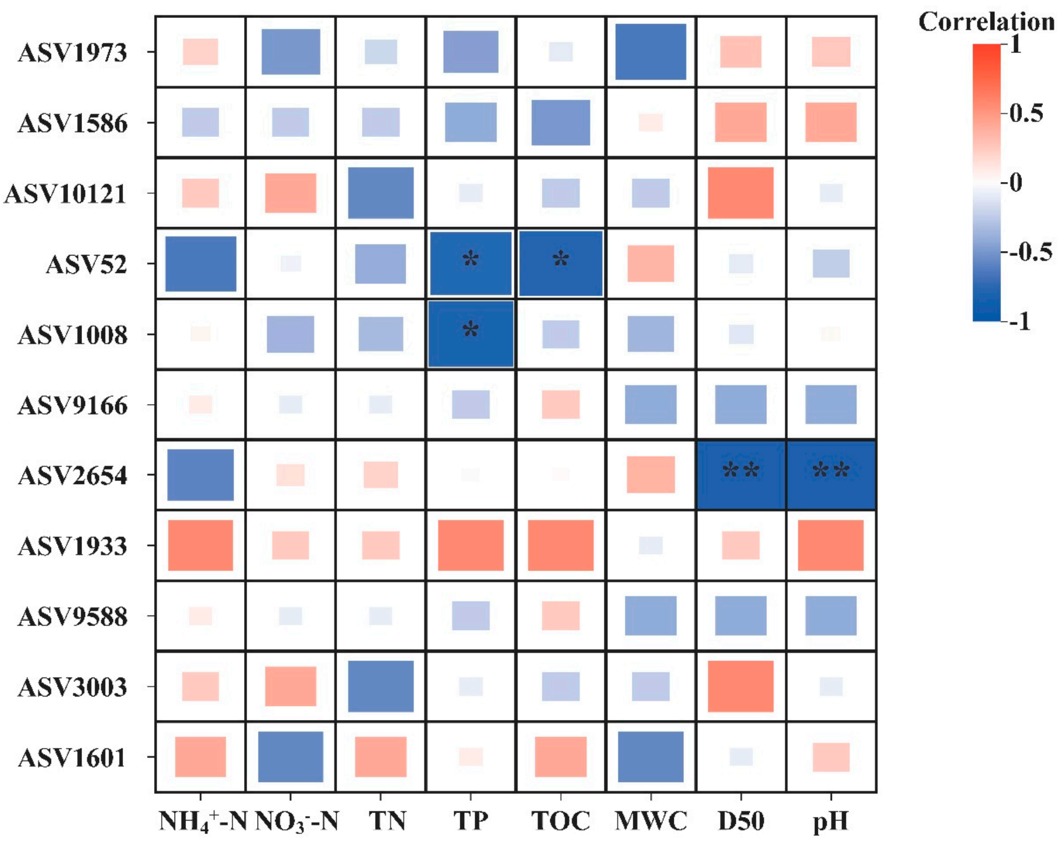

Figure S3. Correlation between functional eukaryotic microorganisms and environmental factors in the GH zone of Lake Taihu (*, $P < 0.05$; **， $P < 0.01$; ***， $P < 0.001$).

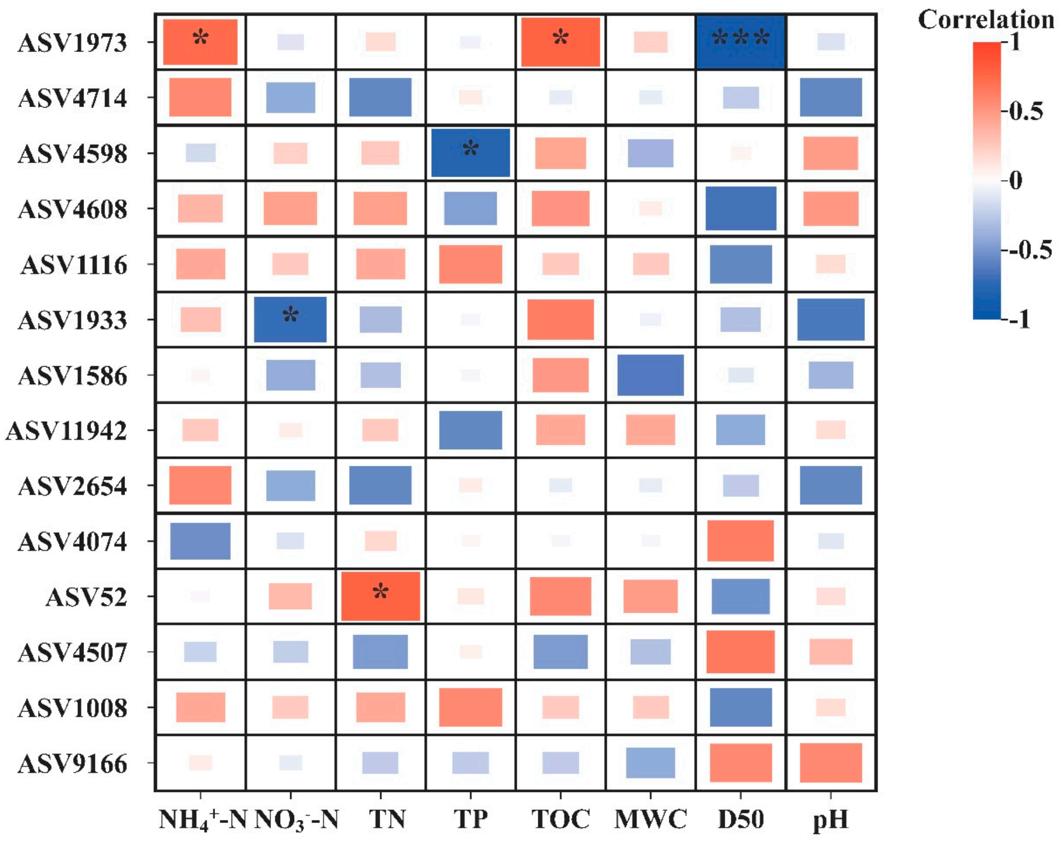

Figure S4. Correlation between functional eukaryotic microorganisms and environmental factors in the HX zone of Lake Taihu (*, $P < 0.05$; **， $P < 0.01$; ***， $P < 0.001$).

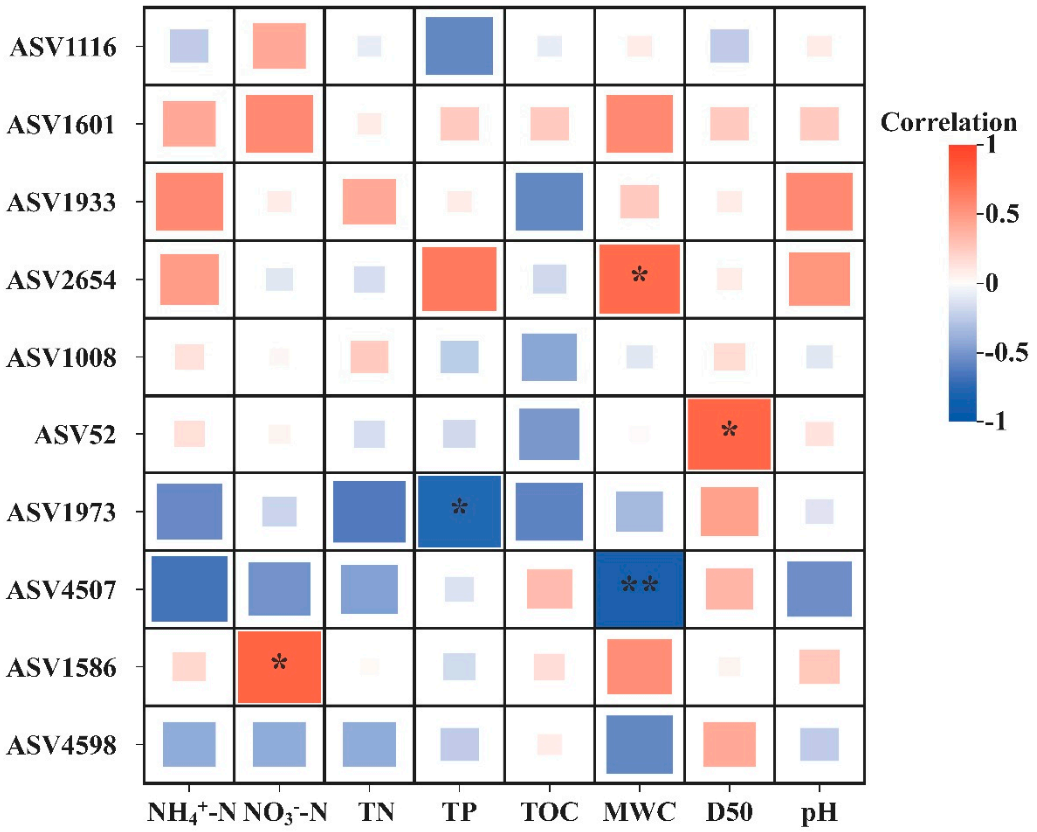

Figure S5. Correlation between functional eukaryotic microorganisms and environmental factors in the XH zone of Lake Taihu (*, $P < 0.05$; **， $P < 0.01$; ***， $P < 0.001$).

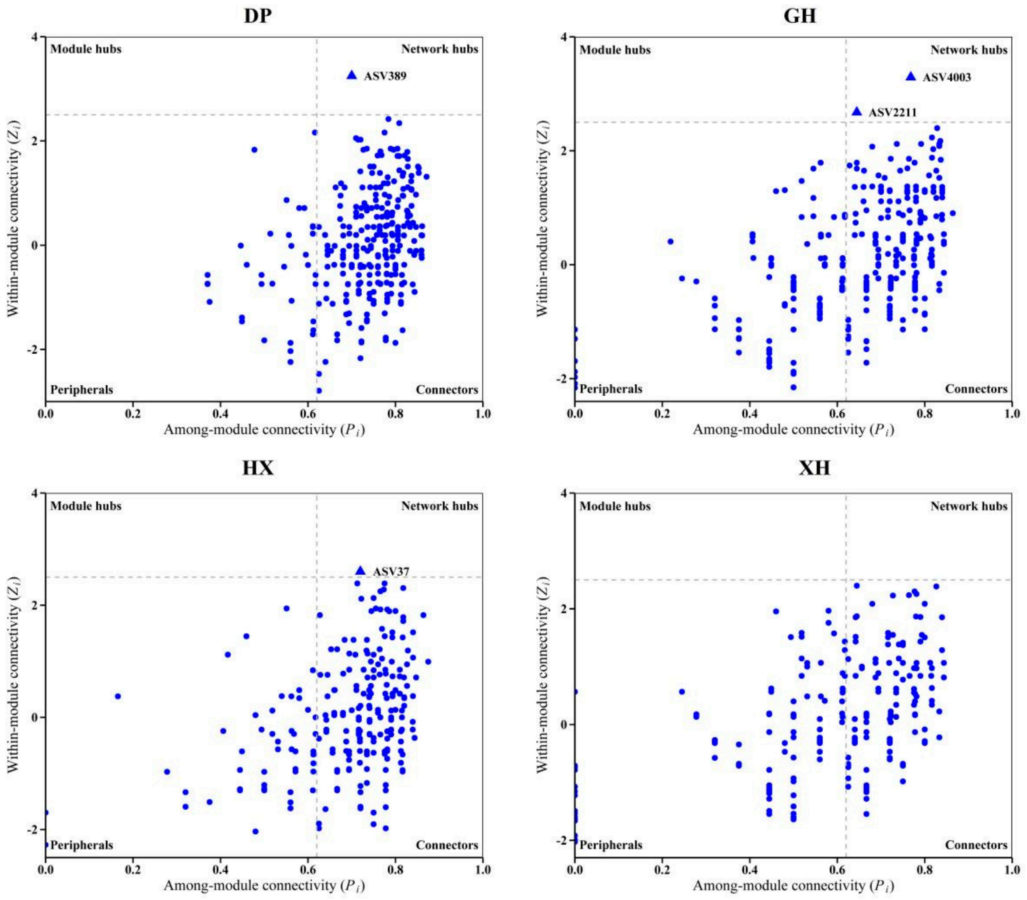

Figure S6. Zi-Pi plots for each network.

Table S1

Sampling sites and their geographic coordinates.

Sampling site	Longitude	Latitude
DP_1	119°57′19.6272"E	31°17'55.5756"N
DP_2	119°56′48.6455"E	31°18′23.3100"N
DP_3	119°57'09.9360”E	31°18′48.2472"N
GH_1	120°08′11.7492”E	31°08′34.2204"N
GH_2	120°08′14.7120”E	31°08′40.4196"N
GH_3	120°08′21.6168”E	31°08′41.9244"N
HX_1	120°20′45.5388”E	31°23′10.0176"N
HX_2	120°21′28.9152"E	31°22′57.2916"N
HX_3	120°21′32.7132”E	31°23′2.7960"N
XH_1	120°24′20.1708”E	31°10′13.9980"N
XH_2	120°24′48.4416”E	31°10′36.6564"N
XH_3	120°24′59.4036”E	31°10′42.2004"N

Note: The number in the Sample ID column indicates the depth of the sedimentary layer.

Table S2

Specific results of environmental factors. Ammonia nitrogen, nitrate nitrogen, total nitrogen, total phosphorus, total organic carbon, mass water content, median particle diameter and pH were abbreviated as NH_4^+-N, NO_3^--N, TN, TP, TOC, MWC, D50 and pH, respectively.

Sample ID	NH_4^+-N (mg/L)	NO_3^--N (mg/L)	TN (mg/L)	TP (mg/L)	TOC (g/kg)	MWC (%)	D50 (μm)	pH
DP1	3.01	5.76	1201.17	973.5	13.76	48.38	11.62	7.41
DP2	5.78	10.51	967.25	1053.24	12.46	42.53	13.2	7.24
DP3	6.95	4.44	616.37	867.52	11.69	40.31	12.34	7.09
DP4	8.23	5.46	628.42	647.28	13.13	46.88	12.85	7.28
DP5	9.06	5.72	502.11	530.41	12.32	42.74	12.1	7.24
DP6	14.82	3.03	513.88	446.42	12.76	44.23	12.65	7.2
DP7	7.31	4.34	820.1	424.43	11.25	43.75	13.41	7.3
DP8	8.94	3.79	982.78	492.22	10.54	42.55	11.76	7.38
GH1	7.28	4.11	1293	644	19.52	54.72	9.82	7.38
GH2	8.82	1.41	991	569	20.02	44.57	10.06	7.5
GH3	10.62	3.29	740	489	18.43	39.77	10.76	7.55
GH4	10.44	1.7	741	530	18.43	35.91	11.02	7.58
GH5	13.19	2.03	798	533	20.1	33.64	9.85	7.49
GH6	18.59	0.63	1152	557	22.27	32.5	10.42	7.55
GH7	18.68	3.59	1062	682	22.73	34.39	10.79	7.59
GH8	18.28	3.87	494	549	18.97	34.15	13.44	7.54
HX1	0.61	5.98	485.17	546.56	8.62	51.65	10.35	7.76
HX2	2.92	2.61	260	505.67	9.92	41.14	11.56	7.82
HX3	2.06	2.53	207.37	621.17	9.62	42.48	9.9	7.7
HX4	2.81	4.71	322.49	487	16.9	40.56	5.73	7.77
HX5	3.12	3.16	365.26	467.51	17.79	43.89	5.22	7.76
HX6	3.18	3.61	427.75	786.78	17.42	42.96	5.18	7.76
HX7	2.11	0.21	281.05	590.97	18.08	41.97	5.79	7.66

HX8	3.6	2.03	102.39	554.37	13.65	42.15	5.52	7.44
XH1	3.77	1.28	140	462	15.8	36.57	21.84	7.32
XH2	3.11	1.98	91	459	14.13	38.12	22.4	7.24
XH3	4.34	4.25	302	428	14.72	45.07	12.96	7.41
XH4	6.2	4.15	582	463	18.3	44.51	10.68	7.24
XH5	6.01	2.46	370	531	18.64	43.79	11.66	7.36
XH6	6.74	4.81	355	510	18.22	51.31	17.21	7.51
XH7	6.24	0.4	163	537	14.09	48.85	13.92	7.64
XH8	8.61	3.53	518	507	13.25	48.77	14.03	7.68

Note: The number in the Sample ID column indicates the depth of the sedimentary layer.

Table S3

Statistical comparison of sediment environmental variables among four depositional zones in Lake Taihu.

Environmental variable	DP	GH	HX	XH	Data presentation	Statistical test	P value	Significance	Significant pairwise comparisons
NH4+-N	7.77 (2.31)	11.90 (8.32)	2.87 (1.04)	6.11 (2.17)	median (IQR)	Kruskal-Wallis	<0.001	***	DP vs. HX; GH vs. HX; GH vs. XH
NO3	4.95 (1.53)	2.66 (2.03)	2.88 (1.48)	3.00 (2.37)	median (IQR)	Kruskal-Wallis	0.026	*	DP vs. GH
TN	779.01 ± 254.38	908.88 ± 261.31	306.44 ± 122.26	315.12 ± 177.76	mean ± sd	One-way ANOVA	<0.001	***	HX-DP; XH-DP; HX-GH; XH-GH
TP	588.85 (413.24)	553.00 (55.50)	550.46 (97.52)	485.00 (54.00)	median (IQR)	Kruskal-Wallis	0.107	ns	
TOC	12.39 (1.27)	19.77 (1.81)	15.27 (7.67)	15.26 (4.12)	median (IQR)	Kruskal-Wallis	<0.001	***	DP vs. GH; GH vs. HX; DP vs. XH
MWC	43.25 (2.35)	35.15 (6.95)	42.31 (1.43)	44.79 (6.42)	median (IQR)	Kruskal-Wallis	0.096	ns	
D50	12.50 (0.92)	10.59 (0.84)	5.76 (4.57)	13.97 (5.73)	median (IQR)	Kruskal-Wallis	<0.001	***	DP vs. HX; GH vs. XH; HX vs. XH

pH	7.26 (0.09)	7.54 (0.06)	7.76 (0.07)	7.38 (0.24)	median (IQR)	Kruskal-Wallis	<0.001	***	DP vs. GH; DP vs. HX; HX vs. XH

Note: Values are expressed as mean ± SD or median (IQR), depending on data distribution and homogeneity of variance. One-way ANOVA or Kruskal–Wallis tests were used for group comparisons, followed by Tukey's HSD or Dunn's post hoc tests, respectively. A significance level of $p < 0.05$ was applied.

Table S4

Vertical variation in alpha diversity indices of sedimentary eukaryotic microbial communities across different depositional zones of Lake Taihu.

Sample	Chao1	Shannon	Simpson
DP1	173	4.500549	0.978585
DP2	244	4.481135	0.973106
DP3	167.3333	4.275646	0.968066
DP4	139.6667	3.798449	0.943545
DP5	77.33333	2.834523	0.773356
DP6	66	2.801218	0.811523
DP7	51.66667	2.816952	0.902157
DP8	108	3.919588	0.959968
GH1	243	2.730565	0.677176
GH2	300	4.675216	0.971191
GH3	248.3333	4.216445	0.942647
GH4	162	3.174173	0.707269
GH5	216.3333	4.491094	0.975556
GH6	193.6667	4.408778	0.97691
GH7	125	3.942878	0.962205
GH8	136.3333	4.127767	0.967813
HX1	244.3333	4.425193	0.967847
HX2	180.3333	3.831967	0.92603
HX3	95.33333	3.748066	0.94376
HX4	42.66667	2.568554	0.846943
HX5	45.66667	2.563432	0.835183
HX6	39.33333	1.694879	0.615563
HX7	56.33333	3.315935	0.938884
HX8	53.66667	2.43982	0.738906
XH1	198	3.34204	0.881321
XH2	79	2.939687	0.856976
XH3	91	3.483668	0.935692
XH4	71.33333	3.017268	0.867693
XH5	112	3.613072	0.925574
XH6	82.66667	3.265596	0.90961
XH7	46	2.743027	0.824613
XH8	63.66667	3.147869	0.912041

Note: The table presents the Chao1, Shannon, and Simpson indices of sedimentary eukaryotic microbial communities across different sediment depths in the DP, GH, HX, and XH zones of Lake Taihu.

Table S5

FUNGuild functional annotation results of fungal ASVs from different sediment depths in the DP depositional zone of Lake Taihu.

ASV ID	DP1	DP2	DP3	DP4	DP5	DP6	DP7	DP8	Taxon	Taxon Level	Trophic Mode	Guild
ASV1973	0	52	21	0	190	6	16761	10	Saccharomycopsis	13	Pathotroph-Saprotroph	Animal Parasite-Undefined Saprotroph
ASV11261	0	0	0	0	0	0	1052	0	Phlebia	13	Saprotroph	Wood Saprotroph
ASV52	0	0	2	18	25	0	534	0	Agaricales	7	Pathotroph-Saprotroph-Symbiotroph	Bryophyte Parasite-Dung Saprotroph-Ectomycorrhizal-Fungal Parasite-Leaf Saprotroph-Plant Parasite-Undefined Saprotroph-Wood Saprotroph
ASV11266	0	0	0	0	0	0	561	0	Candida	13	Pathotroph-Saprotroph-Symbiotroph	Animal Pathogen-Endophyte-Endosymbiont-Epiphyte-Undefined Saprotroph
ASV12957	0	0	0	0	0	0	505	0	Acantharia	13	Pathotroph-Saprotroph	Plant Pathogen-Plant Saprotroph
ASV2654	0	0	0	0	494	0	0	0	Saccharomyces	13	Saprotroph	Undefined Saprotroph
ASV6502	0	0	0	0	0	0	0	373	Morella	13	Pathotroph	Animal Parasite
ASV9166	0	0	0	0	0	0	0	371	Rozella	13	Pathotroph-Symbiotroph	Fungal Parasite
ASV1008	0	0	10	0	0	0	0	3	Pichia	13	Pathotroph-Saprotroph-Symbiotroph	Animal Parasite-Animal Pathogen-Animal Symbiotroph-Plant Pathogen-Undefined Saprotroph

ASV1586	0	0	0	0	0	6	0	0	Malassezia	13	Pathotroph-Saprotroph	Animal Pathogen-Undefined Saprotroph
ASV1116	0	0	0	4	0	0	0	0	Mortierellales	7	Saprotroph-Symbiotroph	Endophyte-Soil Saprotroph-Undefined Saprotroph
ASV4608	0	0	0	0	0	2	0	0	Aspergillus	13	Pathotroph-Saprotroph-Symbiotroph	Animal Pathogen-Endophyte-Plant Saprotroph-Undefined Saprotroph-Wood Saprotroph

Note: This supplementary table integrates the functional annotation results for the DP zone. Rows represent unique annotated fungal ASVs and columns DP1–DP8, indicate sequence abundances in each sample/depth layer. Functional categories include pathotroph, saprotroph, symbiotroph, and mixed trophic modes, supporting the results described in Figure 6.

Table S6

FUNGuild functional annotation results of fungal ASVs from different sediment depths in the GH depositional zone of Lake Taihu.

ASV ID	GH1	GH2	GH3	GH4	GH5	GH6	GH7	GH8	Taxon	Taxon Level	Trophic Mode	Guild
ASV52	103	72	864	53390	122	3	0	4	Agaricales	7	Pathotroph-Saprotroph-Symbiotroph	Bryophyte Parasite-Dung Saprotroph-Ectomycorrhizal-Fungal Parasite-Leaf Saprotroph-Plant Parasite-Undefined Saprotroph-Wood Saprotroph
ASV10121	0	0	0	0	0	0	0	604	Nowakowskiella	13	Pathotroph-Saprotroph	Algal Saprotroph-Plant Pathogen-Plant Saprotroph
ASV9588	0	0	0	0	528	0	0	0	Aurantiochytrium	13	Saprotroph	Plant Saprotroph
ASV1008	0	0	125	31	328	2	0	0	Pichia	13	Pathotroph-Saprotroph-Symbiotroph	Animal Parasite-Animal Pathogen-Animal Symbiotroph-Plant Pathogen-Undefined Saprotroph
ASV3003	0	0	0	0	0	0	0	158	Saccharomycetales	7	Saprotroph	Undefined Saprotroph
ASV1973	0	0	0	107	5	7	0	4	Saccharomycopsis	13	Pathotroph-Saprotroph	Animal Parasite-Undefined Saprotroph
ASV2654	8	3	2	0	61	0	0	0	Saccharomyces	13	Saprotroph	Undefined Saprotroph
ASV1601	0	0	0	0	0	49	0	0	Meyerozyma	13	Saprotroph-Symbiotroph	Endophyte-Epiphyte-Undefined Saprotroph

ASV1586	0	0	0	10	0	0	0	0	Malassezia	13	Pathotroph-Saprotroph	Animal Pathogen-Undefined Saprotroph
ASV1933	0	0	0	0	0	0	7	0	Vishniacozyma	13	Saprotroph	Undefined Saprotroph
ASV9166	0	0	0	0	5	0	0	0	Rozella	13	Pathotroph-Symbiotroph	Fungal Parasite

Note: This supplementary table integrates the functional annotation results for the GH zone. Rows represent unique annotated fungal ASVs and columns GH1–GH8, indicate sequence abundances in each sample/depth layer. Functional categories include pathotroph, saprotroph, symbiotroph, and mixed trophic modes, supporting the results described in Figure 6.

Table S7

FUNGuild functional annotation results of fungal ASVs from different sediment depths in the HX depositional zone of Lake Taihu.

ASV ID	HX1	HX2	HX3	HX4	HX5	HX6	HX7	HX8	Taxon	Taxon Level	Trophic Mode	Guild
ASV1973	0	0	0	8	5002	916	10	248	Saccharomycopsis	13	Pathotroph-Saprotroph	Animal Parasite-Undefined Saprotroph
ASV4714	0	0	0	0	0	0	0	1417	Mucorales	7	Saprotroph	Undefined Saprotroph
ASV4598	28	51	0	438	393	0	259	0	Chytridiaceae	9	Pathotroph-Saprotroph	Algal Parasite-Fungal Parasite-Undefined Saprotroph
ASV4608	0	0	0	434	588	11	0	0	Aspergillus	13	Pathotroph-Saprotroph-Symbiotroph	Animal Pathogen-Endophyte-Plant Saprotroph-Undefined Saprotroph-Wood Saprotroph
ASV1116	0	0	0	0	0	1031	0	0	Mortierellales	7	Saprotroph-Symbiotroph	Endophyte-Soil Saprotroph-Undefined Saprotroph
ASV1933	0	0	0	0	7	0	505	214	Vishniacozyma	13	Saprotroph	Undefined Saprotroph
ASV1586	0	0	0	201	0	0	427	5	Malassezia	13	Pathotroph-Saprotroph	Animal Pathogen-Undefined Saprotroph
ASV11942	0	0	0	0	594	0	0	0	Saccharomycopsis	13	Pathotroph-Saprotroph	Animal Parasite-Undefined Saprotroph
ASV2654	0	0	0	0	0	0	0	586	Saccharomyces	13	Saprotroph	Undefined Saprotroph

ASV4074	23	18	0	0	0	0	430	0	Paramicrosporidium	13	Pathotroph	Protist Parasite
ASV52	43	18	20	39	70	105	65	0	Agaricales	7	Pathotroph-Saprotroph-Symbiotroph	Bryophyte Parasite-Dung Saprotroph-Ectomycorrhizal-Fungal Parasite-Leaf Saprotroph-Plant Parasite-Undefined Saprotroph-Wood Saprotroph
ASV4507	0	140	92	0	0	0	0	0	Chytridiaceae	9	Pathotroph-Saprotroph	Algal Parasite-Fungal Parasite-Undefined Saprotroph
ASV1008	0	0	0	0	0	4	0	0	Pichia	13	Pathotroph-Saprotroph-Symbiotroph	Animal Parasite-Animal Pathogen-Animal Symbiotroph-Plant Pathogen-Undefined Saprotroph
ASV9166	0	4	0	0	0	0	0	0	Rozella	13	Pathotroph-Symbiotroph	Fungal Parasite

Note: This supplementary table integrates the functional annotation results for the HX zone. Rows represent unique annotated fungal ASVs and columns HX1–HX8, indicate sequence abundances in each sample/depth layer. Functional categories include pathotroph, saprotroph, symbiotroph, and mixed trophic modes, supporting the results described in Figure 6.

Table S8

FUNGuild functional annotation results of fungal ASVs from different sediment depths in the XH depositional zone of Lake Taihu.

ASV ID	XH1	XH2	XH3	XH4	XH5	XH6	XH7	XH8	Taxon	Taxon Level	Trophic Mode	Guild
ASV1008	0	10	0	6	0	0	0	2515	Pichia	13	Pathotroph-Saprotroph-Symbiotroph	Animal Parasite-Animal Pathogen-Animal Symbiotroph-Plant Pathogen-Undefined Saprotroph
ASV1973	42	501	1168	0	0	0	8	11	Saccharomycopsis	13	Pathotroph-Saprotroph	Animal Parasite-Undefined Saprotroph
ASV52	17	109	0	0	0	41	0	425	Agaricales	7	Pathotroph-Saprotroph-Symbiotroph	Bryophyte Parasite-Dung Saprotroph-Ectomycorrhizal-Fungal Parasite-Leaf Saprotroph-Plant Parasite-Undefined Saprotroph-Wood Saprotroph
ASV1933	0	0	0	0	0	0	0	314	Vishniacozyma	13	Saprotroph	Undefined Saprotroph
ASV2654	0	0	0	0	0	58	230	0	Saccharomyces	13	Saprotroph	Undefined Saprotroph
ASV4507	119	50	0	0	69	0	0	0	Chytridiaceae	9	Pathotroph-Saprotroph	Algal Parasite-Fungal Parasite-Undefined Saprotroph
ASV1116	0	0	70	0	0	0	0	0	Mortierellales	7	Saprotroph-Symbiotroph	Endophyte-Soil Saprotroph-Undefined Saprotroph
ASV1586	0	0	12	0	0	38	0	0	Malassezia	13	Pathotroph-Saprotroph	Animal Pathogen-Undefined Saprotroph
ASV4598	17	0	0	0	0	0	0	0	Chytridiaceae	9	Pathotroph-Saprotroph	Algal Parasite-Fungal Parasite-Undefined Saprotroph

ASV1601	0	0	0	0	0	15	0	0	Meyerozyma	13	Saprotroph-Symbiotroph	Endophyte-Epiphyte-Undefined Saprotroph

Note: This supplementary table integrates the functional annotation results for the XH zone. Rows represent unique annotated fungal ASVs and columns XH1–XH8, indicate sequence abundances in each sample/depth layer. Functional categories include pathotroph, saprotroph, symbiotroph, and mixed trophic modes, supporting the results described in Figure 6.

Table S9

Topological parameters of microbial co-occurrence networks in four depositional zones of Lake Taihu.

Parameter	DP	GH	HX	XH
Num.Nodes	332	396	263	315
Num.Edges	1878	1385	1224	903
Num.Communities	10	12	10	12
Average.Degree	11.313	6.995	9.308	5.733
Avg.Weighted.Degree	2.798	1.641	2.28	1.353
Network.Diameter	4	6	5	7
Graph.Density	0.034	0.018	0.036	0.018
Modularity	0.294	0.373	0.324	0.419
Avg.Clustering.Coefficient	0.054	0.042	0.058	0.029
Avg.Path.length	2.662	3.319	2.746	3.503

Note: The table summarizes the main topological parameters of the co-occurrence networks constructed for the DP, GH, HX, and XH zones.
